# An App-Based Cognitive Behavioral Therapy Program Tailored for College Students: Randomized Controlled Trial

**DOI:** 10.2196/50006

**Published:** 2025-06-19

**Authors:** Min Hee Kim, Yeon-kug Moon, Kyong-Mee Chung

**Affiliations:** 1 Department of Psychology, Yonsei University, 50 Yonsei-ro, Seodaemun-gu, Seoul, Republic of Korea, 82 221232448; 2 Department of Artificial Intelligence and Data Science, Sejong University, Seoul, Republic of Korea

**Keywords:** t-CBT, college students, depressive symptoms, randomized controlled trial, RCT, app-based, cognitive behavioral therapy, CBT, self-help, technology-based cognitive behavioral therapy, mobile phone

## Abstract

**Background:**

Technology-based cognitive behavioral therapy programs are accessible interventions to address mental health challenges, particularly among college students. Despite their potential, low adherence rates remain a key challenge.

**Objective:**

This study aims to assess the effects of the tailored content and gamification elements of the Mind Booster Green program on reducing depressive symptoms and improving college life adjustment.

**Methods:**

A randomized, unblinded controlled trial was conducted among college students. All study procedures were conducted remotely using web-based tools. The participants were randomly assigned to the intervention or waitlist control groups. The intervention group used Mind Booster Green, an app-based self-help cognitive behavioral therapy program for 1 month. The program included tailored case stories and gamification elements, such as point and level systems, to enhance user engagement and adherence. Outcomes were self-assessed using web-based questionnaires and included changes in depressive symptoms, college life adjustment, and negative and positive automatic thoughts. The usability of the app was also evaluated. Outcomes were measured at 3 time points: preintervention, postintervention, and at a 2-month follow-up (F/U), using validated and standardized tools.

**Results:**

A total of 170 individuals (mean age 22.60, SD 3.37 years; 136/170, 80% female) participated in the study. A chi-square analysis revealed no significant differences between the two groups at baseline in terms of age, sex, or class year (*P*>.05). A generalized estimating equation analysis revealed significant time×group interactions for all variables. Compared to the control group, the intervention group showed greater improvements across all outcomes, with between-group effect sizes ranging from -0.78 to 0.49. For derpessive symptoms, large within-group effect size were observed (Patient Health Questionnaire-9: pre to post, Cohen *d*=1.12; pre to F/U, Cohen *d*=1.15; Beck Depression Inventory-II: pre to post, Cohen *d*=0.90; pre to F/U, Cohen *d*=1.04). Large within-group effect size was also found for adjustment to college life (Student Adaptation to College Questionnaire–Revised: pre to post, Cohen *d*=–0.87; pre to F/U, Cohen *d*=-0.85), and moderate effect for negative automatic thoughts (Automatic Thought Questionnaire–Negative, Short Form: pre to post, Cohen *d*=0.36; pre to F/U, Cohen *d*=0.58) and positive automatic thoughts (Automatic Thought Questionnaire—Positive, Short Form: pre to post, Cohen *d*=–0.45; pre to F/U, Cohen *d*=-0.44). Adherence rates were 89% and 99% for the intervention and control groups, respectively. The usability test results, assessed using the Mobile App Rating Scale, showed an overall score of 3.88, with scores above the medium level in the engagement, functionality, aesthetics, and information quality categories.

**Conclusions:**

Mind Booster Green demonstrated substantial potential as a complementary interventio to traditional psychological services for college students, providing a cost-effective and scalable solution for mental health issues. Future research should explore the applicability of this program in diverse populations.

## Introduction

Cognitive behavioral therapy (CBT) is an evidence-based treatment with substantial evidence supporting its effectiveness in alleviating depressive symptoms [1]. However, delivery constraints, such as requiring 12‐16 weeks of face-to-face treatment by a professional, have hindered its widespread dissemination [2]. Recent advances in digital technology, including smartphones, have led to the development of technology-based CBT (t-CBT) programs that use digital tools. Several studies have demonstrated the effectiveness of these programs in reducing depressive symptoms [3-6]. With its advantages, such as high accessibility, low cost, and minimal stigmatization, t-CBT is gaining attention as an innovative treatment delivery method that can supplement or even provide an alternative to traditional face-to-face CBT [7].

However, participation in t-CBT interventions relies on users’ willingness and motivation, resulting in the need to address issues such as low engagement and high dropout rates. For example, meta-analyses have reported user adherence rates of only 17%‐26% [8,9], and usage surveys of real-world open-access unguided programs have reported much lower rates of approximately 4% [10]. The degree of adherence to an intervention program can be linked to its effectiveness [11]; therefore, the need to consider and research strategies to improve usability, user engagement, and adherence is increasing [12].

Tailoring has recently been proposed as a strategy for enhancing user engagement and adherence. This strategy involves personalizing an intervention’s content and layout based on users’ demographic and psychological characteristics and use context [13]. Tailoring is particularly suitable for diagnostic groups with heterogeneous symptoms or demographic factors, such as individuals with depression, and is achieved by clearly defining the treatment target group based on specific factors (eg, age, occupation, socioeconomic status, and ethnicity) and developing appropriate treatment strategies accordingly [14,15]. Tailored content can provide personally relevant information directly to users, thereby increasing the initial uptake and use of an app and facilitating changes in user attitudes or behaviors [16]. In the context of t-CBT, tailoring strategies can be approached in several ways, including (1) providing content that reflects the characteristics and experiences of the target population [17-20], (2) developing personalized treatment plans according to individuals’ diagnoses or symptoms [21,22], and (3) enabling users to customize features such as menus, tools, and notifications [23]. Tailored mental health intervention programs have been generally reported to positively affect user adherence, thereby enhancing program effectiveness and user satisfaction [16,24,25].

Among the aspects of tailoring, providing content that reflects the characteristics and experiences of the target population is suggested to facilitate users’ acquisition of emotional, cognitive, and behavioral skills without expert assistance [26,27]. In practice, t-CBT uses targeted audience-specific examples, language, and various multimedia formats (eg, text, video, and pictures) to deliver psychoeducational content, providing the necessary information and modeling of skills they need to learn to be delivered to users more effectively [24]. For example, Strecher et al [20] found that providing tailored fictional success stories reflecting user demographics such as sex, age, race, marital status, and smoking-related factors increased long-term engagement in a smoking cessation program, which was linked to program effectiveness.

Gamification, which involves applying game elements to nongame digital platforms (such as web- or app-based systems) [28], is another strategy for encouraging user engagement and adherence. Gamification has been reported to increase user motivation and engagement, make interactions with app-based programs enjoyable, and positively influence behavioral changes related to physical health and psychological well-being [29,30]. In technology-based mental health interventions, gamification is suggested to play a role in increasing app use and user engagement and supporting the delivery of the intervention’s active ingredients, thereby enhancing therapeutic outcomes [31]. In a previous meta-analysis, Cheng and Ebrahimi [32] showed that gamified interventions are more effective than nongamified ones in reducing depressive and anxiety symptoms. In the context of t-CBT for mental health, gamification has been implemented using avatars [33,34], storytelling, and reward systems [35,36]. Specifically, several self-management program studies have shown that positive reinforcement such as points, badges, digital rewards, and tangible prizes have been shown to effectively promote health-related target behaviors [37]. These strategies must be considered for active applications in t-CBT to encourage users to log in regularly, use the app, and complete in-app activities [38].

Although t-CBT has primarily been developed and deployed in populations with specific psychiatric conditions, interest in its application in younger populations, particularly college students, is growing. Depression is a common mental health concern among college students [39,40] and can interfere with academic retention [41], career development [42], and social relationships [43], ultimately affecting their overall quality of life [44]. The increasing prevalence of depression among college students has become a social concern for clinicians and mental health professionals [45,46]. Institutional efforts, such as student counseling services, aim to address students’ mental health needs; however, actual usage rates are low because of long wait times and concerns about social stigma [47-50]. In this context, t-CBT may serve as a useful means of addressing such difficulties. Given college students’ high smartphone penetration and favorable attitudes toward app-based programs [51,52], t-CBT holds significant potential for improving access to mental health care and addressing the unmet needs of this population.

Building on the existing research, two strategies can be applied to t-CBT programs aimed at reducing depressive symptoms among college students. First, programs should be tailored to the cognitive, behavioral, and emotional characteristics of depressive symptoms experienced by college students, as well as stressors related to college life. Second, positive reinforcement strategies should be incorporated through gamification to encourage college students to use t-CBT programs voluntarily. This is based on research findings that younger digital natives [53], such as current college students, are more likely to find gamified products useful and enjoyable [54]; moreover, achievement-related gamification features, including badges and upgrades, have a more positive impact on this group than on older groups [55].

Despite its potential, relatively few studies have focused on developing and validating t-CBT programs that incorporate tailoring or gamification to alleviate depression among college students. While several studies have adapted interventions originally designed for the general adult populations [56-58], such efforts often lack sufficient consideration for the distinctive psychological and contextual needs of college students. Nevertheless, some studies have indicated the potential of tailored t-CBT programs for this population. Mullin et al [18] demonstrated significant reductions in depressive and anxiety symptoms among college students using a tailored therapist-guided CBT program (UniWellbeing). Similarly, Lattie et al [59] reported increased mental health literacy and high use rates among students who used a tailored, unguided CBT program (IntelliCare). Recent evidence from an Asian context supports the effectiveness of internet-based CBT programs tailored for local college students, further suggesting their broader applicability [60]. In Korea, some CBT programs originally developed for adults have been evaluated among college students [61]; however, such studies remain limited. Furthermore, studies incorporating gamification elements that are explicitly designed to enhance voluntary engagement and sustained use are scarce, indicating a notable gap and underscoring the need for additional research.

To address this gap, this study developed and evaluated the effectiveness of Mind Booster Green, a tailored, app-based self-help CBT program for reducing depressive symptoms among college students. Changes in depressive symptoms and adaptation to college life were assessed to verify the effectiveness of the program. Additionally, changes in negative and positive automatic thoughts were examined to verify the applicability of CBT mechanisms to students. App usability was evaluated using standardized measures of engagement, functionality, aesthetics, and information quality.

## Methods

### Recruitment

Participants were recruited through advertisements on social media (eg, Facebook and Naver) and web-based communities popular among college students and young adults (eg, Everytime and job search cafés). Individuals had to meet the following inclusion criteria: (1) undergraduate or graduate students; (2) native Korean speakers with no difficulty using smartphones; (3) used a smartphone with iOS 12 or higher or Android 8 or higher; (4) scored 5 or higher on the Patient Health Questionnaire-9 (PHQ-9), indicating mild depression, and less than 1 on the suicide item, indicating a low risk of suicide; and (5) were not currently receiving other mental health services (eg, medication, psychotherapy, or counseling). The participants received an explanation of the study through a video posted on the study website and signed an electronic consent form. Additionally, under institutional review board (IRB) approval, they submitted a copy of their ID to verify their identity.

### Study Setting

The study was conducted remotely using web-based tools in South Korea from June 2021 to March 2022. All study procedures, including recruitment, screening, consent, intervention delivery, and outcome assessments, were conducted remotely owing to the impact of COVID-19. Data were collected through Qualtrics (Qualtrics), and survey links were delivered individually via a dedicated social networking service (SNS) channel. All participant communications, including survey timing notifications, survey links, reminders, inquiries, and risk issues, were managed through this channel. All procedures were performed by trained researchers as listed in the IRB-approved protocol.

### Measures

#### PHQ-9

The Korean version of the PHQ-9, developed by Kroenke et al [62] and validated by Park et al [63], was used to screen participants and measure changes in depression severity. The PHQ-9 consists of 9 items that assess depressive symptoms experienced over the past 2 weeks, rated on a 4-point Likert scale from 0=not at all to 3=nearly every day. It was administered during the screening to identify participants with mild or higher levels of depression (PHQ-9≥5) and no moderate or higher suicidal ideation (PHQ-9 item 9≤1). Given the time gap between screening and baseline assessments, the PHQ-9 was also included in the pre, post, and follow-up assessments. The internal consistency (Cronbach ɑ) in the Korean standardization study [64] was 0.95, and in this study, it was 0.79.

#### Korean Beck Depression Inventory-II

The Korean version of the Beck Depression Inventory-II (BDI-II), developed by Beck et al [65] and validated by Kim et al [66], was used to assess changes in depression severity. The BDI-II is a 21-item standardized questionnaire that is widely used to evaluate depressive symptoms. Participants rated their level of difficulty with depressive symptoms such as sadness, lethargy, feelings of worthlessness, and changes in sleep and appetite over the past 2 weeks on a 4-point Likert scale ranging from 0=not at all to 3=severely. Regarding internal consistency, Cronbach ɑ was 0.83 in the Korean standardization study [67] and 0.89 in this study.

#### Automatic Thought Questionnaire—Negative/Positive, Short Form

The Automatic Thought Questionnaire—Negative, Short Form (ATQN-SF) and the Automatic Thought Questionnaire—Positive, Short Form (ATQP-SF) were used to assess participants’ patterns of automatic thoughts—both negative and positive. The ATQN-SF, developed by Hollon and Kendall [68], adapted into Korean by Kwon and Yoon [69], and further refined by Heo and Kim [70], comprises 9 items rated on a 5-point Likert scale (0=not at all to 4=always). It measures negative automatic thoughts associated with depression and assesses changes in thought patterns in alignment with CBT mechanisms. The ATQP-SF, a 10-item scale adapted and validated in Korean by Heo and Kim [71] from the original Automatic Thought Questionnaire-Revised by Kendall et al [72], evaluates the frequency of positive automatic thoughts, which are often lacking in individuals vulnerable to depression. Both subscales demonstrated excellent internal consistency, with Cronbach ɑ values of 0.96 (ATQN-SF) and 0.93 (ATQP-SF) reported in previous studies and 0.89 (ATQN-SF) and 0.91 (ATQP-SF) in this study.

#### Student Adaptation to College Questionnaire—Revised

The Student Adaptation to College Questionnaire—Revised (SACQ-R) was used to measure participants’ adaptation to college life. Developed by Baker and Siryk [73] and revised and adapted into Korean by Lee [74], the SACQ-R assesses academic, social, emotional, and physical adjustment, as well as attachment to the institution. The 25-item measure is rated on a 5-point Likert scale ranging from 1=not at all to 5=very much. Regarding internal consistency, Cronbach ɑ was 0.85 in previous research [74] and in this study.

#### Mobile App Rating Scale

To evaluate the usability of the app, the Mobile App Rating Scale (MARS) developed by Stoyanov et al [75] was translated into Korean and used according to the guidelines of the International Test Commission [76]. The scale comprises 33 items across five factors: engagement, functionality, aesthetics, information quality, and app-specific items (subjective satisfaction). Participants rated each item on a 5-point Likert scale ranging from 1=not at all to 5=very much. Additionally, an open-ended question was included to gather unstructured subjective feedback: “What did you like about using the app?” Usability was assessed postintervention in the intervention group (n=80).

### Design and Procedure

#### Overview

All study procedures were conducted in accordance with the CONSORT-EHEALTH (Consolidated Standards of Reporting Trials of Electronic and Mobile Health Applications and Online Telehealth) guidelines. This was a two-arm, parallel-group, randomized controlled trial with a waitlist control (WLC) design. An a priori power analysis was conducted using a linear multiple regression model (*F* test) in G*Power (Heinrich Heine University Düsseldorf), as no standardized procedure is available for power analysis in generalized estimating equation (GEE). The analysis indicated that a minimum sample size of 42 participants was required to detect a medium effect size (*F*_2_=0.25) with *P*=.05 and power=0.80, assuming two predictors (df1=2, df2=39), based on prior t-CBT efficacy studies.

Participants who provided informed consent were randomly assigned to the intervention or WLC group. Randomization was conducted using a pregenerated random number table created in Excel (Microsoft Corp) by a research assistant registered in the IRB-approved protocol. The assistant used the =RAND() function to generate random values, which were sorted to assign the participants to groups. As this was an open-label trial, both the participants and investigators were aware of the group assignments.

Participants who met the inclusion criteria underwent the following steps: (1) preintervention assessment, (2) intervention or waiting period depending on group assignment, (3) postintervention assessment, and (4) follow-up assessment. The preintervention assessment was conducted immediately after participants provided informed consent. The postintervention assessment was conducted after the intervention group completed the 28-session Mind Booster Green training and 30 days postbaseline for the WLC group. A follow-up assessment was conducted 2 months after the postintervention assessment. Both groups completed the PHQ-9, BDI-II, SACQ-R, ATQN-SF, and ATQP-SF at all the assessment points. Additionally, the MARS was administered to the intervention group during the postintervention assessment for usability evaluation.

After completing the 2-month follow-up assessment, participants in the WLC group were granted access to the app. App access was provided only after all required assessments were completed. No symptom data or app use records were collected from the WLC group following the intervention, and postintervention data were not included in the outcome analyses.

#### Intervention Condition: Mind Booster Green Group

Participants assigned to the intervention group downloaded and installed the app from Apple’s app store (iOS) or the Google Play store (Android) via a URL provided by the researchers. Upon registration, the researchers approved the use of the app on the admin page. The participants began training independently without additional guidance or support and were advised to complete one session per day, with the researchers monitoring their progress via the admin page. Participants who did not log in or complete sessions for more than 3 days received prompt SMS text messages, and those who did not engage for more than 10 days were considered withdrawn. Other than the SMS text message prompts to resume the use of the app, participants received no feedback on their performance from the researchers, who were only available to answer questions about the study via an SNS channel.

#### Mind Booster Green

Mind Booster Green is a self-help CBT app adapted from “HaruToday,” a CBT app initially developed for patients with cancer [17,77] with the support of the Korean Ministry of Health and Welfare (HA16C0021). HaruToday has three modules (depression or anxiety, sleep, and pain issues) and has been validated for its effectiveness on depression or anxiety [17] and sleep [77]. The Mind Booster Green program was modified to address depression in undergraduate and graduate students by reducing the number of sessions from 48 to 28 based on user feedback. Tailored CBT content was developed through focus group interviews with undergraduate and graduate students to gather specific case stories related to their experiences with depression. A pilot test involving CBT experts and college students was conducted, and the final version was completed through revision and supplementation. The program used in this study was not updated during the experimental period [78].

The main training program comprises 5 zones with 28 sessions targeting effective CBT techniques for managing depressive symptoms: (1) psychoeducation, (2) cognitive restructuring, (3) behavioral activation, (4) relaxation training, and (5) problem-solving. Each zone includes 4‐10 sessions, each lasting approximately 10‐15 minutes and consisting of 50‐70 illustrated slides with pages where users can input answers, complete quizzes, or fill out worksheets. Audio narration is available for each slide and can be customized by the user.

**Figure 1.**
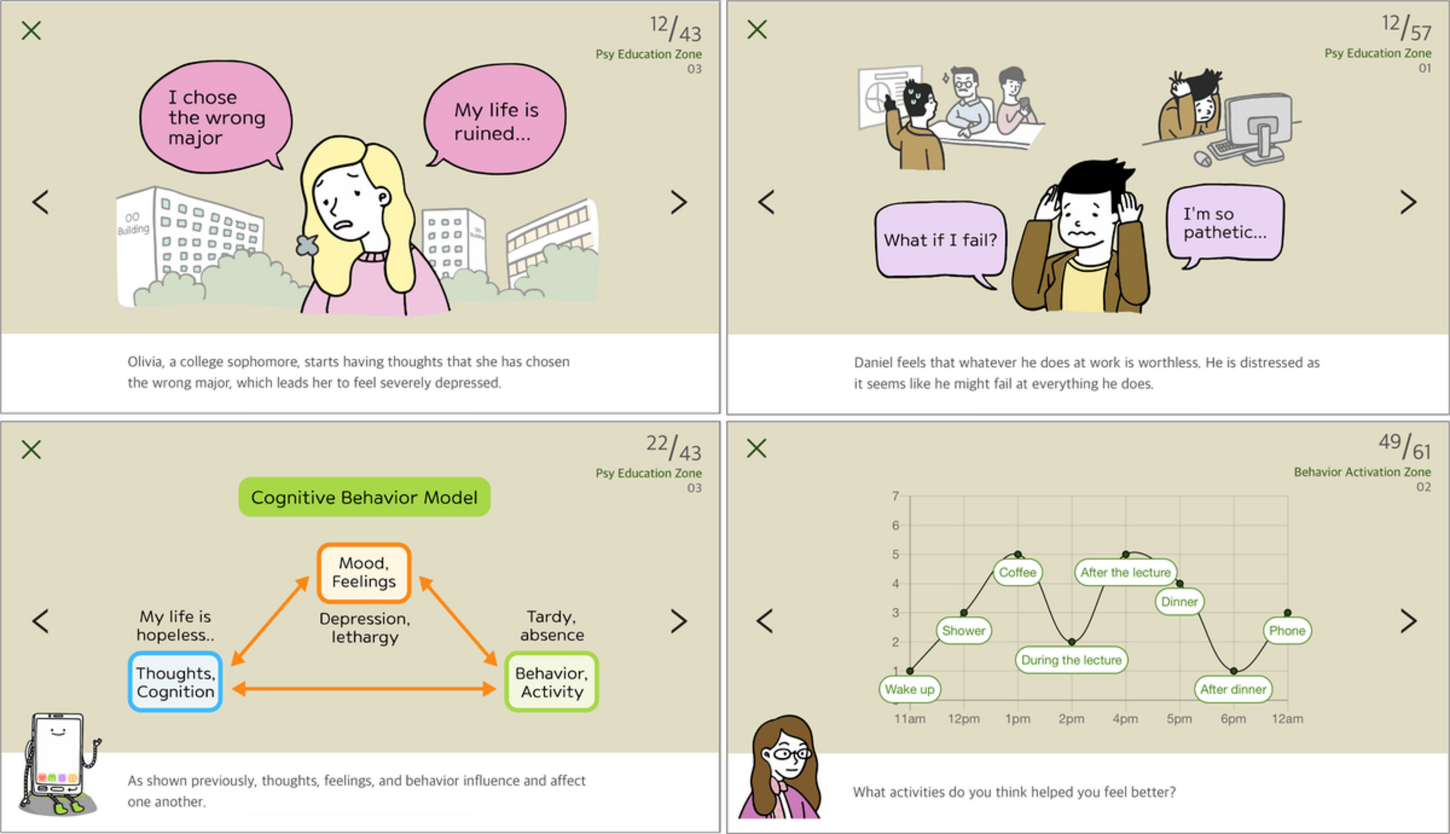
Example screen from Mind Booster Green.

Six characters representing college students are featured in the story and the text and illustrations detail how they work with experts to explore their depressive symptoms, learn cognitive-behavioral skills to manage their symptoms, and overcome challenges (Figure 1). Users also engage in self-guided tasks such as quizzes and activity planning to apply learned skills. Table 1 summarizes the specific characteristics of each zone. The app includes mood-monitoring features, allowing users to track their mood visually and providing thought records and relaxation audio or video to apply the learned techniques in real life.

**Table 1.** Session outline of Mind Booster Green.

Training zone	Number of sessions	Content
Psychoeducation	4	Characteristics of depressive symptoms Core principles of CBT^a^ Self-monitoring techniques
Behavioral activation	5	Pleasant activities exploration Pleasant activities planning
Cognitive restructuring	10	Automatic thoughts and cognitive distortions Cognitive restructuring techniques Thought record
Problem-solving	5	Five-step problem-solving approach Time management Assertiveness skills Anger management strategies
Relaxation	4	Progressive muscle relaxation Guided imagery techniques

a CBT: cognitive behavioral therapy.

Mind Booster Green uses point-and-level systems based on positive reinforcement. Participants earned points for attendance and completion of mood checks, sessions, training zones, and thought records. Accumulating points led to leveling, with 5 levels requiring progressively more points. As the participants leveled up, the main character’s potted plant grew, providing encouragement and emotional support.

#### WLC Condition

Participants in the WLC group were instructed to maintain their usual activities for 30 days without additional guidance or support from the researchers. Similar to the intervention group, they were able to contact the research team through the SNS channel for study-related inquiries.

### Statistical Analysis

The analyses included data from 91 participants in the intervention group, 79 participants in the control group for the intention-to-treat (ITT) analysis, and 78 participants in both groups for the per-protocol (PP) analysis. One-way ANOVAs and chi-square analyses were conducted to examine demographic characteristics and ensure group homogeneity in the preassessment. To evaluate the effectiveness of the intervention, an ITT analysis [79] was performed using the entire dataset, including dropouts, and a PP analysis was conducted with participants who completed all procedures. A GEE was used to test for group differences in changes over time [80]. As all dependent variables were nonnormally distributed, the GEE analysis used an unstructured working correlation structure and gamma distribution with a log-link response scale. Time and group were the predictor variables, and the dependent variables included the PHQ-9, BDI-II, SACQ-R, ATQN-SF, and ATQP-SF, with preassessment scores as covariates. Statistical analyses were performed using SPSS (version 27; IBM Corp). Within- and between-group effect sizes (Cohen *d*) were calculated using absolute scores at each time point.

### Ethical Considerations

This study was reviewed and approved by the IRB of Yonsei University (IRB 7001988‐202202HR-1127-10) in accordance with the institutional and national ethical guidelines for research involving human participants. All participants were fully informed about the purpose, procedures, and voluntary nature of the study through a video explanation posted on the study website and provided written informed consent by electronically signing a digital consent form prior to participation. They were also informed of their right to withdraw from the study at any time, without penalty. All collected data were deidentified to ensure the participants’ privacy and confidentiality. No personally identifiable information was stored, and all data were securely managed on encrypted servers with access limited to authorized research staff. Participants received monetary compensation equivalent to 50,000 KRW (approximately US $40) after completing the follow-up assessment, with prorated compensation for partial participation. Participants in the WLC group were granted access to the app after the follow-up assessment, along with compensation. These measures were implemented to ensure fairness and acknowledge the participants’ time and contributions to the study.

### Trial Registration

This study was approved by the IRB prior to its initiation. However, the clinical trial was retrospectively registered in the Korean Clinical Trial Registry (Clinical Research Information Service) under registration number KCT0009758. At the time of the study, the necessity of prospective clinical trial registration was not fully recognized, particularly because trial registration is not a legal requirement in Korea. However, we promptly addressed this oversight and retrospectively completed the registration. The research procedures reported in the registration are fully consistent with the protocol approved by the IRB. It was ensured that all research procedures adhered to the ethical standards approved by the IRB and that retrospective registration did not impact the integrity or outcomes of the study.

## Results

### Participant Flow and Sample Description

**Figure 2.**
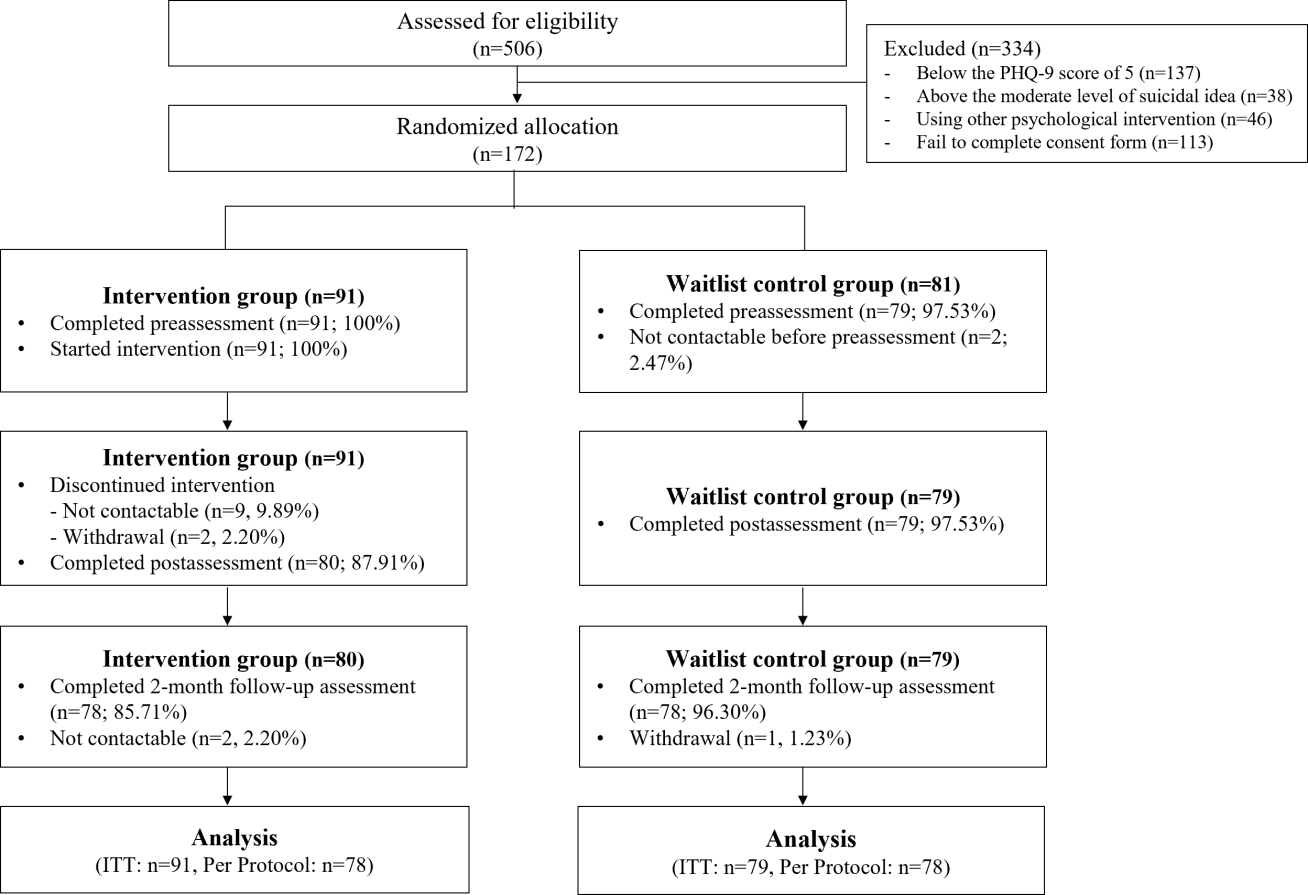
CONSORT diagram of participant flow. CONSORT: Consolidated Standards of Reporting Trials; ITT: intention-to-treat; PHQ-9: Patient Health Questionnaire-9.

Of the 506 undergraduate and graduate students who volunteered to participate, 172 met the inclusion criteria and were enrolled. A pregenerated random number table in Excel was used to randomly assign participants to the intervention (n=91) or WLC (n=81) group. Before the baseline assessment, 2 participants in the WLC group ceased contact and were excluded from the study. During the intervention period, 11 participants were excluded owing to loss of contact (intervention group, n=9) or voluntary withdrawal (intervention group, n=2). Between the postintervention and follow-up assessments, contact with 2 participants from the intervention group was lost, and 1 participant from the WLC group was excluded because they used other psychological services. Consequently, 156 participants (78 in each group) completed all three assessments. For the ITT analysis, data from 91 and 79 participants in the intervention and control groups, respectively, were included. For the PP analysis, data from 78 participants in both groups were included. Figure 2 presents a detailed flowchart of the participant selection process.

The mean age of the study participants (n=170) was 22.60 (SD 3.37, median 22, IQR 18‐38) years, with 80% (136/170) female and 20% (34/170) male participants. Of the total participants, 17.1% (29/170) were freshmen, 17.6% (30/170) sophomores, 24.1% (41/170) juniors, 22.9% (39/170) seniors or above, and 18.2% (31/170) graduate students. Table 2 shows the demographic characteristics of participants in both groups. The chi-square analysis showed no differences between the intervention and WLC groups in terms of age, sex, or class year (Table 2; all *P*>.05).

**Table 2.** Participant characteristics by group.

Characteristics		Intervention (n=91)	Control(n=79)	Chi-square (*df*)	*P* value
Age (years), mean (SD)		22.31 (3.34)	22.91 (3.41)	1.31^a^ (1, 168)	.25
Sex, n (%)				0.0 (1)	.94
Female		73 (80.2)	63 (79.7)		
Male		18 (19.8)	16 (20.3)	
Year, n (%)				3.3 (4)	.51
Freshman		14 (15.4)	15 (19.0)		
Sophomore		20 (22.0)	10 (12.7)	
Junior		19 (20.9)	22 (27.8)	
Senior level or above		21 (23.1)	18 (22.8)	
Graduate student		17 (18.7)	14 (17.7)	

a For age, *F* test value is provided.

Table 3 presents the observed means and standard deviations of the PHQ-9, BDI-II, SACQ-R, ATQP-SF, and ATQN-SF scores by group at preassessment and the results of the between-group analyses of variance. No significant differences were observed between the groups for any variable at preassessment (*P*>.05).

**Table 3.** Observed means and SDs for scores of each variable by group.

Measures and time		Intervention (Pre n=91; Post n=80; F/U^a^ n=78), mean (SD)	Control (Pre n=79; Post n=79; F/U n=78), mean (SD)	*F* test (*df*)	*P* value
PHQ-9^b^				0.98 (1, 168)	.40
Pre		10.75 (4.12)	10.13 (4.00)
Post		6.09 (4.19)	8.51 (4.57)		
F/U		5.81 (4.43)	7.46 (4.68)		
BDI-II^c^				2.55 (1, 168)	.14
Pre		22.59 (9.51)	20.23 (9.01)		
Post		13.38 (10.80)	17.90 (9.73)		
F/U		12.26 (10.65)	16.21 (10.62)		
SACQ-R^d^				0.84 (1, 168)	.33
Pre		68.13 (12.40)	69.91 (12.97)		
Post		79.16 (13.05)	71.76 (16.98)		
F/U		79.38 (14.17)	73.27 (16.83)		
ATQP-SF^e^				1.40 (1, 168)	.40
Pre		12.67 (6.23)	13.85 (6.76)		
Post		15.85 (7.83)	14.28 (7.56)		
F/U		16.18 (9.52)	14.71 (9.36)		
ATQN-SF^f^				2.49 (1, 168)	.10
Pre		11.51 (7.77)	9.75 (6.60)		
Post		8.66 (7.89)	9.75 (8.48)		
F/U		7.24 (6.99)	9.42 (7.98)		

a F/U: follow-up.

b PHQ-9: Patient Health Questionnaire-9.

c BDI-II: Beck Depression Inventory-II.

d SACQ-R: Student Adaptation to College Questionnaire—Revised.

e ATQP-SF: Automatic Thought Questionnaire—Positive, Short Form.

f ATQN-SF: Automatic Thought Questionnaire—Negative, Short Form.

### Effectiveness of Mind Booster Green

#### ITT Analysis

**Figure 3.**
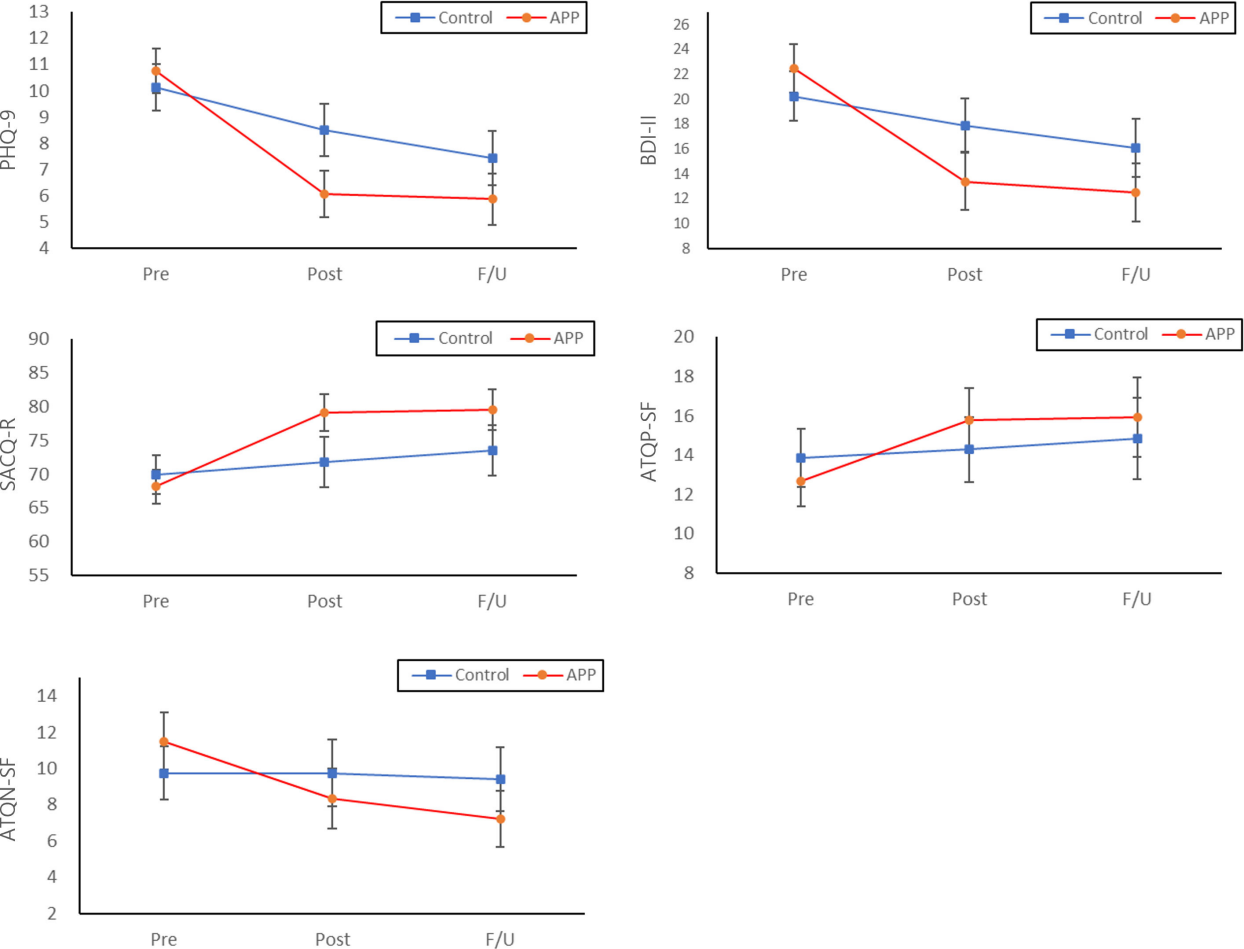
Means and 95% intervals for the groups across the assessment time points. APP: ____; ATQN-SF: Automatic Thought Questionnaire—Negative, Short Form; ATQP-SF: Automatic Thought Questionnaire—Positive, Short Form; BDI-II: Beck Depression Inventory-II; PHQ-9: Patient Health Questionnaire-9; SACQ-R: Student Adaptation to College Questionnaire—Revised.

The estimated marginal means and SEs of the PHQ-9, BDI-II, SACQ-R, ATQP-SF, and ATQN-SF scores at the pre, post, and follow-up assessments are presented in Table 4, and the trends in score changes across time points are illustrated in Figure 3. The GEE analysis revealed a significant time×group interaction for all variables, and the main effects of group and time were significant for all 5 variables. These findings indicated significant differences between the intervention and control groups in terms of changes in depressive symptoms, adjustment to college life, and positive and negative automatic thoughts over time. The paired comparisons showed that the intervention group had lower scores for depressive symptoms (PHQ-9 and BDI-II) and negative automatic thoughts (ATQN-SF) and higher scores for adjustment to college life (SACQ-R) and positive automatic thoughts (ATQP-SF) after the intervention than before the use of Mind Booster Green. An examination of the effect size of the change from pre- to postassessment and follow-up assessment scores in the intervention group showed a large decrease in depressive symptom scores after using the app (pre to post Cohen *d*: PHQ-9 1.12, BDI-II 0.90), followed by a small but sustained decrease in scores up to 2 months after the postassessment (pre to F/U Cohen *d*: PHQ-9 1.15, BDI-II 1.04). Negative automatic thoughts followed a pattern similar to that of depressive symptoms; however, the magnitude of change was smaller (pre to post Cohen *d*=0.36; pre to F/U Cohen *d*=0.58). Although scores for adjustment to college life and positive automatic thoughts increased at postassessment compared with preassessment (pre to post Cohen *d*: SACQR –0.87, ATQP-SF –0.45), little difference was found in these scores between the post and follow-up assessments (pre to F/U Cohen *d*: SACQ-R –0.85, ATQP-SF –0.44). Table 5 presents the effect sizes of the mean score changes at each time point.

**Table 4.** Results of the GEE^a^ analysis with covariate adjustment for the preassessment scores of each variable^b^.

Measures		Intention-to-treat		Per protocol	
		Wald chi-square (*df*)	*P* value	Wald chi-square (*df*)	*P* value
PHQ-9^c^					
Group×Time		28.7 (2)	<.001	29.5 (2)	<.001
Group		17.0 (1)	<.001	18.1 (1)	<.001
Time		118.1 (2)	<.001	118.0 (2)	<.001
BDI-II^d^					
Group×Time		28.5 (2)	<.001	35.8 (2)	<.001
Group		23.1 (1)	<.001	30.6 (1)	<.001
Time		81.8 (2)	<.001	96.0 (2)	<.001
SACQ-R^e^					
Group×Time		30.9 (2)	<.001	32.0 (2)	<.001
Group		26.2 (1)	<.001	27.5 (1)	<.001
Time		93.3 (2)	<.001	94.9 (2)	<.001
ATQP-SF^f^					
Group×Time		7.6 (2)	.02	7.8 (2)	.02
Group		6.0 (1)	.02	5.9 (1)	.02
Time		35.9 (2)	<.001	35.8 (2)	<.001
ATQN-SF^g^					
Group×Time		9.3 (2)	.01	9.0 (2)	.01
Group		10.3 (1)	.001	10.0 (1)	.002
Time		2.8 (2)	.24	2.8 (2)	.25

a GEE: generalized estimating equation.

b Intervention pre n=91, post n=80, F/U n=78; Control pre n=79, post n=79, F/U n=78.

c PHQ-9: Patient Health Questionnaire-9.

d BDI-II: Beck Depression Inventory-II.

e SACQ-R: Student Adaptation to College Questionnaire—Revised.

f ATQP-SF: Automatic Thought Questionnaire—Positive, Short Form.

g ATQN-SF: Automatic Thought Questionnaire—Negative, Short Form.

**Table 5.** Estimated marginal means and effect size.

	Preintervention (n=91), Control (n=79)			Postintervention (n=80), Control (n=79)			F/U^a^ intervention (n=78), Control (n=78)			Within-group effect size from prescores				Between-group effect size			
										Post		F/U		Post		F/U	
	Mean (SE^b^)		95% CI^c^	Mean(SE)		95% CI	Mean(SE)		95% CI	Cohen *d*	95% CI	Cohen *d*	95% CI	Cohen *d*	95% CI	Cohen *d*	95% CI
PHQ-9^d^														–0.78	–1.22 to –0.34	–0.36	–0.68 to –0.05
Intervention	9.84(0.08)		9.68 to 10.00	5.68(0.32)		5.09 to 6.34	5.96(0.49)		5.08 to 7.00	1.12	0.82 to 1.42	1.15	0.85 to 1.46				
Control	9.71(0.10)		9.51 to 9.91	8.32(0.42)		7.53 to 9.20	7.38(0.41)		6.61 to 8.23	0.38	0.07 to 0.69	0.61	0.30 to 0.93				
BDI-II^e^														–0.44	–0.75 to –0.13	–0.37	–0.69 to –0.06
Interventio	19.87(0.18)		19.52 to 20.23	11.87(0.80)		10.4 to 13.55	11.18(0.89)		9.57 to 13.06	0.90	0.60 to 1.2	1.04	0.71 to 1.37				
Control	19.66(0.32)		19.04 to 20.29	17.62(0.75)		16.21 to 19.16	16.26(0.90)		14.60 to 18.12	0.25	–0.06 to 0.56	0.41	0.10 to 0.72				
SACQ-R^f^														0.49	0.18 to 0.80	0.39	0.08 to 0.71
Intervention	67.64(0.28)		67.09 to 68.20	79.54(1.31)		77.01 to 82.14	80.21(1.57)		77.2 to 83.34	–0.87	–1.17 to –0.57	–0.85	–1.15 to –0.54				
Control	67.82(0.33)		67.17 to 68.48	70.68(0.93)		68.87 to 72.53	71.94(1.55)		68.96 to 75.05	–0.12	–0.43 to 0.19	–0.22	–0.54 to 0.09				
ATQP-SF^g^														0.20	–0.11 to 0.51	0.16	–0.16 to 0.47
Intervention	11.81(0.22)		11.39 to 12.25	15.68(0.68)		14.4 to 17.08	16.04(0.86)		14.43 to 17.81	–0.45	–0.75 to –0.15	–0.44	–0.74 to –0.13				
Control	11.83(0.21)		11.42 to 12.24	13.00(0.73)		11.64 to 14.52	13.52(0.82)		12.01 to 15.21	–0.06	–0.37 to 0.25	–0.10	–0.42 to 0.21				
ATQN-SF^h^														–0.13	–0.44 to 0.18	–0.29	–0.60 to 0.02
Intervention	9.01(0.26)		8.51 to 9.55	7.56(0.55)		6.55 to 8.71	6.86(0.64)		5.72 to 8.23	0.36	0.06 to 0.66	0.58	0.28 to 0.88				
Control	9.04(0.27)		8.53 to 9.58	9.41(0.59)		8.33 to 10.63	9.85(0.76)		8.46 to 11.47	0.00	–0.31 to 0.31	0.04	–0.27 to 0.36				

a F/U: follow-up.

b SE: Standard Error

c CI: Confidence Interval

d PHQ-9: Patient Health Questionnaire-9.

e BDI-II: Beck Depression Inventory-II.

f SACQ-R: Student Adaptation to College Questionnaire–Revised.

g ATQP-SF: Automatic Thought Questionnaire—Positive, Short Form.

h ATQN-SF: Automatic Thought Questionnaire–Negative, Short Form.

The control group showed a decrease in depressive symptoms from pre- to postassessment and follow-up; however, the magnitude was smaller than that in the intervention group, and the group mean scores were higher than those in the intervention group when comparing the PHQ-9 and BDI-II scores for the post and follow-up assessments (Table 5). When comparing scores for the pre, post, and follow-up assessments, the effect sizes (Cohen *d*) for changes in adjustment to college life and positive and negative automatic thoughts ranged from −0.22 to 0.04, indicating negligible or a small change.

#### PP Analysis

A PP analysis was conducted on the data of participants who completed all 28 sessions of the Mind Booster Green training program and all three assessments. Adherence rates were 89% and 99% in the intervention and WLC groups, respectively. The analysis was conducted using the GEE, as in the ITT analysis. The results showed significant time×group interactions for the PHQ-9, BDI-II, SACQ-R, ATQP-SF, and ATQN-SF scores and significant main effects of group and time for all variables. These findings were consistent with those of the ITT analysis. The results of the PP analysis are presented in Table 4.

### Usability Test Results

The usability test was conducted using the MARS in the intervention group (n=80). The average usability score for Mind Booster Green was 3.88. Across domains, the mean scores were 3.57 (SD 0.66) for engagement, 4.17 (SD 0.76) for functionality, 3.40 (SD 0.75) for aesthetic quality, 4.36 (SD 0.56) for information quality, and 3.92 (SD 0.67) for app-specific features. All domain scores were above the medium level.

## Discussion

### Principal Findings

Mind Booster Green is an app-based CBT program designed to alleviate depressive symptoms in college students. To enhance the program’s effectiveness and encourage continuous and independent use, the following strategies were applied: (1) tailoring the content using case stories reflecting the experiences college students are likely to have and (2) incorporating gamification elements, such as point and level systems, to promote app use. To verify the effectiveness of the program, we examined the reduction in depressive symptoms and improvement in college life adjustment and assessed the app’s usability using a standardized measure. The key findings and implications of this study were as follows.

First, the self-help t-CBT program Mind Booster Green was found to be effective in alleviating depressive symptoms in college students immediately after completing the training provided by the app, and the effect was sustained for at least 2 months postintervention. Additionally, the intervention group that used Mind Booster Green reported improved academic, social, emotional, and health-related adjustment to college life. These results are encouraging as they demonstrate that app-based CBT programs can effectively reduce depressive symptoms through cognitive-behavioral skills training and extend these improvements to students’ real-life functioning. The results of this study suggest that Mind Booster Green can complement or serve as an alternative to existing psychological counseling services, providing a cost-effective and efficient solution to mental health issues among college students.

Second, based on a detailed examination of the program’s effectiveness, the intervention group exhibited reductions in depressive symptoms and changes in automatic thoughts, specifically a decrease in negative automatic thoughts and an increase in positive thoughts. Although this study did not conduct a formal mediation analysis to determine whether changes in thought patterns directly mediated symptom improvement, these findings are consistent with the theoretical underpinnings of CBT that emphasize the role of cognitive restructuring in alleviating emotional distress. CBT assumes that emotions and behaviors are influenced by individuals’ cognitive appraisals and seeks to reduce psychological symptoms by identifying and modifying negatively biased thoughts through cognitive and behavioral strategies [81]. Acquiring skills to challenge and replace maladaptive automatic thoughts is considered a core component of CBT treatment [82,83]. Researchers have emphasized the importance of embedding these core mechanisms into digital CBT interventions and delivering them in ways that maintain therapeutic integrity [12]. The observed changes in automatic thoughts suggest that the program successfully incorporated the key elements of CBT and may have contributed to symptom improvement. Although relatively few studies have directly examined therapeutic mechanisms in t-CBT interventions, existing evidence reflects cognitive changes that are in line with CBT theory, suggesting potential applicability across different interventions [84-87]. These findings support the fundamental theoretical model of CBT and demonstrate its effectiveness across digital formats. They also provide empirical support for the active implementation of t-CBT as a practical and scalable approach for treating depression.

Third, despite being a self-help program, Mind Booster Green showed a relatively high adherence rate. Mind Booster Green was designed for students to use independently without the help of therapists or experts. Although self-help programs maximize the economic and accessibility advantages of t-CBT programs, low adherence rates remain a challenge. The adherence rate of 89% in the intervention group was significantly higher than the reported adherence rate of approximately 26% for technology-based self-help interventions but similar to the 85% adherence rate reported for traditional face-to-face interventions [9]. Although adherence rates in randomized controlled trials are generally higher than those in observational studies [88,89], the rate observed in this study is notable. Enhancing user engagement and participation in intervention programs is closely linked to effectiveness [11], making adherence an important issue to address when developing self-help t-CBT programs. Thus, this study actively applied two strategies—tailoring and gamification—and the high adherence rate indirectly suggests their effectiveness.

The tailoring strategy in Mind Booster Green was designed based on social learning theory and intended to effectively model the cognitive and behavioral strategies of CBT. Although tailoring can be broadly applied to provide information that fits user characteristics and needs, the tailoring strategy in Mind Booster Green focuses on program content composition. The program features college students who share their experiences related to depression and learn CBT skills to overcome their problems, thereby modeling how users in similar situations can apply these skills in their lives. Specific narratives reflecting the characteristics of a particular population facilitate effective modeling, a technique widely used in various psychological therapy contexts [90] and reported to enhance user engagement [91]. The changes in automatic thoughts observed alongside the reduction in depressive symptoms in this study indicated that the cognitive skills targeted by the program were appropriately conveyed to the participants. Additionally, approximately 20% of the study participants mentioned that “real college student cases” in the content helped them relate to the program and learn CBT skills.

The gamification strategy in Mind Booster Green was designed based on behavioral theory to ensure that users continuously engaged in and completed the app’s treatment activities. Mind Booster Green clarified the tasks that the participants needed to accomplish each day (goal setting), provided feedback on progress (progress and feedback), and implemented point and level systems (rewards) based on task completion. These are the key gamification strategies used in technology-based mental health interventions [92]. Notably, the gamification strategy used in this study was based on behavioral theory and aimed to help users learn and engage in new behaviors. Specifically, we analyzed the tasks that users needed to complete in Mind Booster Green and designed positive reinforcement strategies to increase the performance of those tasks. Points were awarded for engaging in and completing the training tasks, and a level system was introduced that showed the main character evolving as the points accumulated. Behavioral theory scientifically explores the factors that elicit and maintain human behavior, and strategies based on this theory have been reported to be effective in increasing app use behaviors [30,93]. As demonstrated in this study, using task analysis and reinforcement schedules based on established behavioral theories in app-based mental health programs can effectively encourage and teach desired behaviors.

The usability evaluation results for Mind Booster Green also reflected the effectiveness of these strategies. In this study, the MARS [75], a standardized assessment tool, was used to measure usability, allowing for comparisons with other programs. Lau et al [94] used MARS to assess the released app-based mental health interventions and reported average overall scores of 3.52 (SD 0.71). The overall score for Mind Booster Green was 3.88, which was slightly higher than the previously reported results obtained using MARS, with notably higher scores for functionality (4.17) and information quality (4.36). These positive user evaluations suggested that the intervention program development strategy, which considers user characteristics and preferences, positively influenced the user experience. This study clearly defined the target behaviors that users need to acquire to alleviate depressive symptoms and guided these behaviors by developing specific elements of tailoring and gamification based on psychological theory. The effectiveness of Mind Booster Green, the high adherence rate, and positive usability evaluation results highlight the importance of developing theoretically grounded strategies for therapeutic mechanisms and app-use behavior.

### Limitations and Future Directions

This study was limited to one country, the Republic of Korea, and primarily included students from 4-year universities in Seoul, despite recruiting participants from various universities and graduate schools nationwide. The participant demographics showed a sex imbalance, with significantly fewer male participants than female participants. Additionally, the study included a broad range of participants, from freshmen to graduate students. Future studies should include a more diverse range of school types and recruit a larger number of participants of different sexes and class-year groups for repeated verification. Expanding the verification to an international scope to explore whether the program’s effectiveness can be generalized to college students in other countries could also provide valuable data for developing tailored t-CBT programs for the target population.

This study applied tailoring and gamification features based on behavioral theories such as social learning and positive reinforcement to a t-CBT program and showed positive results in reducing depressive symptoms and improving adherence rates and usability. However, future research must investigate how the individual factors of tailoring and gamification mediate these positive outcomes. Existing studies analyzing the impact of these features on program effectiveness and adherence have produced conflicting results [95] owing to the limited number of studies and differences in how these features are defined. Thus, the continuous development of t-CBT programs incorporating tailoring and gamification is needed, along with research on how these effects are mediated by the specific designs and theoretical underpinnings of these strategies.

### Conclusions

This study found that the self-help t-CBT program Mind Booster Green can be effective in reducing depressive symptoms and improving adjustment to college life among college students. These results suggested the potential of self-help t-CBT programs in alleviating and preventing depression in this target group. Future research should test the effectiveness and usability of the strategies applied in this study, including tailoring and gamification, across diverse populations.

## Supplementary material

10.2196/50006 Checklist 1 CONSORT-EHEALTH (Consolidated Standards of Reporting Trials of Electronic and Mobile Health Applications and Online Telehealth) checklist.

## Abbreviations

ATQN-SF: Automatic Though Questionnaire—Negative, Short Form
ATQP-SF: Automatic Though Questionnaire—Positive, Short Form
BDI-II: Beck Depression Inventory-II
CBT: cognitive behavioral therapy
CONSORT-EHEALTH: Consolidated Standards of Reporting Trials of Electronic and Mobile Health Applications and Online Telehealth
GEE: generalized estimating equation
IRB: institutional review board
ITT: intention-to-treat
MARS: Mobile App Rating Scale
PHQ-9: Patient Health Questionnaire-9
PP: per-protocol
SACQ-R: Student Adaptation to College Questionnaire-Revised
SNS: social networking service
t-CBT: technology-based cognitive behavioral therapy
WLC: waitlist control

## References

[1] Cuijpers P, Smit F, Bohlmeijer E, Hollon SD, Andersson G (2010). Efficacy of cognitive–behavioural therapy and other psychological treatments for adult depression: meta-analytic study of publication bias. Br J Psychiatry.

[2] Williams C, Martinez R (2008). Increasing access to CBT: stepped care and CBT self-help models in practice. Behav Cogn Psychother.

[3] Firth J, Torous J, Nicholas J (2017). The efficacy of smartphone-based mental health interventions for depressive symptoms: a meta-analysis of randomized controlled trials. World Psychiatry.

[4] Karyotaki E, Riper H, Twisk J (2017). Efficacy of self-guided internet-based cognitive behavioral therapy in the treatment of depressive symptoms. JAMA Psychiatry.

[5] Saad A, Bruno D, Camara B, D’Agostino J, Bolea-Alamanac B (2021). Self-directed technology-based therapeutic methods for adult patients receiving mental health services: systematic review. JMIR Ment Health.

[6] Edge D, Watkins ER, Limond J, Mugadza J (2023). The efficacy of self-guided internet and mobile-based interventions for preventing anxiety and depression—a systematic review and meta-analysis. Behav Res Ther.

[7] Webb CA, Rosso IM, Rauch SL (2017). Internet-based cognitive-behavioral therapy for depression: current progress and future directions. Harv Rev Psychiatry.

[8] Karyotaki E, Kleiboer A, Smit F (2015). Predictors of treatment dropout in self-guided web-based interventions for depression: an “individual patient data” meta-analysis. Psychol Med.

[9] Richards D, Richardson T (2012). Computer-based psychological treatments for depression: a systematic review and meta-analysis. Clin Psychol Rev.

[10] Baumel A, Edan S, Kane JM (2019). Is there a trial bias impacting user engagement with unguided e-mental health interventions? A systematic comparison of published reports and real-world usage of the same programs. Transl Behav Med.

[11] Donkin L, Christensen H, Naismith SL, Neal B, Hickie IB, Glozier N (2011). A systematic review of the impact of adherence on the effectiveness of e-therapies. J Med Internet Res.

[12] Mohr DC, Burns MN, Schueller SM, Clarke G, Klinkman M (2013). Behavioral intervention technologies: evidence review and recommendations for future research in mental health. Gen Hosp Psychiatry.

[13] Dugas M, Gao GG, Agarwal R (2020). Unpacking mHealth interventions: a systematic review of behavior change techniques used in randomized controlled trials assessing mHealth effectiveness. Digital Health.

[14] Cuijpers P, Karyotaki E, Eckshtain D (2020). Psychotherapy for depression across different age groups: a systematic review and meta-analysis. JAMA Psychiatry.

[15] Toyomoto R, Sakata M, Yoshida K (2023). Prognostic factors and effect modifiers for personalisation of internet-based cognitive behavioural therapy among university students with subthreshold depression: a secondary analysis of a factorial trial. J Affect Disord.

[16] Morrison LG (2015). Theory-based strategies for enhancing the impact and usage of digital health behaviour change interventions: a review. Digital Health.

[17] Ham K, Chin S, Suh YJ (2019). Preliminary results from a randomized controlled study for an app-based cognitive behavioral therapy program for depression and anxiety in cancer patients. Front Psychol.

[18] Mullin A, Dear BF, Karin E (2015). The UniWellbeing course: a randomised controlled trial of a transdiagnostic internet-delivered cognitive behavioural therapy (CBT) programme for university students with symptoms of anxiety and depression. Internet Interventions.

[19] Weaver A, Zhang A, Xiang X, Felsman P, Fischer DJ, Himle JA (2023). Entertain Me Well: an entertaining, tailorable, online platform delivering CBT for depression. Cogn Behav Pract.

[20] Strecher VJ, McClure JB, Alexander GL (2008). Web-based smoking-cessation programs: results of a randomized trial. Am J Prev Med.

[21] Karyotaki E, Klein AM, Ciharova M (2022). Guided internet-based transdiagnostic individually tailored cognitive behavioral therapy for symptoms of depression and/or anxiety in college students: a randomized controlled trial. Behav Res Ther.

[22] Batterham PJ, Calear AL, Farrer L, McCallum SM, Cheng VWS (2018). *FitMindKit*: randomised controlled trial of an automatically tailored online program for mood, anxiety, substance use and suicidality. Internet Interventions.

[23] Balaskas A, Schueller SM, Cox AL, Doherty G (2021). The functionality of mobile apps for anxiety: systematic search and analysis of engagement and tailoring features. JMIR Mhealth Uhealth.

[24] Mukhiya SK, Wake JD, Inal Y, Pun KI, Lamo Y (2020). Adaptive elements in internet-delivered psychological treatment systems: systematic review. J Med Internet Res.

[25] Stawarz K, Preist C, Tallon D, Wiles N, Coyle D (2018). User experience of cognitive behavioral therapy apps for depression: an analysis of app functionality and user reviews. J Med Internet Res.

[26] Cuijpers P, Muñoz RF, Clarke GN, Lewinsohn PM (2009). Psychoeducational treatment and prevention of depression: the “Coping with Depression” course thirty years later. Clin Psychol Rev.

[27] Rigabert A, Motrico E, Moreno-Peral P (2020). Effectiveness of online psychological and psychoeducational interventions to prevent depression: systematic review and meta-analysis of randomized controlled trials. Clin Psychol Rev.

[28] Deterding S, Dixon D, Khaled R, Nacke L (2011). From game design elements to gamefulness: defining “gamification".

[29] Cheng VWS, Davenport T, Johnson D, Vella K, Hickie IB (2019). Gamification in apps and technologies for improving mental health and well-being: systematic review. JMIR Ment Health.

[30] Sardi L, Idri A, Fernández-Alemán JL (2017). A systematic review of gamification in e-Health. J Biomed Inform.

[31] Cheng VWS (2020). Recommendations for implementing gamification for mental health and wellbeing. Front Psychol.

[32] Cheng C, Ebrahimi OV (2023). A meta-analytic review of gamified interventions in mental health enhancement. Comput Human Behav.

[33] Six SG, Byrne KA, Aly H, Harris MW (2022). The effect of mental health app customization on depressive symptoms in college students: randomized controlled trial. JMIR Ment Health.

[34] Fleming TM, Stasiak K, Moselen E (2019). Revising computerized therapy for wider appeal among adolescents: youth perspectives on a revised version of SPARX. Front Psychiatry.

[35] Bakker D, Kazantzis N, Rickwood D, Rickard N (2018). Development and pilot evaluation of smartphone-delivered cognitive behavior therapy strategies for mood- and anxiety-related problems: MoodMission. Cogn Behav Pract.

[36] Miloff A, Marklund A, Carlbring P (2015). The challenger app for social anxiety disorder: new advances in mobile psychological treatment. Internet Interventions.

[37] Devan H, Farmery D, Peebles L, Grainger R (2019). Evaluation of self-management support functions in apps for people with persistent pain: systematic review. JMIR Mhealth Uhealth.

[38] Mullins JK, Sabherwal R (2020). Gamification: a cognitive-emotional view. J Bus Res.

[39] Auerbach RP, Mortier P, Bruffaerts R (2018). WHO World Mental Health Surveys International College Student Project: prevalence and distribution of mental disorders. J Abnorm Psychol.

[40] Chang JJ, Ji Y, Li YH, Pan HF, Su PY (2021). Prevalence of anxiety symptom and depressive symptom among college students during COVID-19 pandemic: a meta-analysis. J Affect Disord.

[41] Awadalla S, Davies EB, Glazebrook C (2020). A longitudinal cohort study to explore the relationship between depression, anxiety and academic performance among Emirati university students. BMC Psychiatry.

[42] Saunders DE, Peterson GW, Sampson JP, Reardon RC (2000). Relation of depression and dysfunctional career thinking to career indecision. J Vocat Behav.

[43] Whitton SW, Whisman MA (2010). Relationship satisfaction instability and depression. J Fam Psychol.

[44] Mahmoud JSR, Staten RT, Hall LA, Lennie TA (2012). The relationship among young adult college students’ depression, anxiety, stress, demographics, life satisfaction, and coping styles. Issues Ment Health Nurs.

[45] Lipson SK, Lattie EG, Eisenberg D (2019). Increased rates of mental health service utilization by U.S. college students: 10-year population-level trends (2007-2017). Psychiatr Serv.

[46] Copeland WE, McGinnis E, Bai Y (2021). Impact of COVID-19 pandemic on college student mental health and wellness. J Am Acad Child Adolesc Psychiatry.

[47] Auerbach RP, Alonso J, Axinn WG (2016). Mental disorders among college students in the World Health Organization World Mental Health Surveys. Psychol Med.

[48] Xiao H, Carney DM, Youn SJ (2017). Are we in crisis? National mental health and treatment trends in college counseling centers. Psychol Serv.

[49] Hunt J, Eisenberg D (2010). Mental health problems and help-seeking behavior among college students. J Adolesc Health.

[50] Raunic A, Xenos S (2008). University counselling service utilisation by local and international students and user characteristics: a review. Int J Adv Counselling.

[51] Poushter J (2016). Smartphone ownership and internet usage continues to climb in emerging economies. https://www.pewresearch.org/global/2016/02/22/smartphone-ownership-and-internet-usage-continues-to-climb-in-emerging-economies.

[52] Batterham PJ, Calear AL (2017). Preferences for internet-based mental health interventions in an adult online sample: findings from an online community survey. JMIR Ment Health.

[53] Creighton TB (2018). Digital natives, digital immigrants, digital learners: an international empirical integrative review of the literature. Educ Leadersh Rev.

[54] Bittner JV, Shipper J (2014). Motivational effects and age differences of gamification in product advertising. J Consum Mark.

[55] Zhang L, Shao Z, Li X, Feng Y (2021). Gamification and online impulse buying: the moderating effect of gender and age. Int J Inf Manage.

[56] Powell J, Hamborg T, Stallard N (2013). Effectiveness of a web-based cognitive-behavioral tool to improve mental well-being in the general population: randomized controlled trial. J Med Internet Res.

[57] Fitzpatrick KK, Darcy A, Vierhile M (2017). Delivering cognitive behavior therapy to young adults with symptoms of depression and anxiety using a fully automated conversational agent (Woebot): a randomized controlled trial. JMIR Ment Health.

[58] Twomey C, O’Reilly G (2017). Effectiveness of a freely available computerised cognitive behavioural therapy programme (MoodGYM) for depression: meta-analysis. Aust N Z J Psychiatry.

[59] Lattie E, Cohen KA, Winquist N, Mohr DC (2020). Examining an app-based mental health self-care program, intellicare for college students: single-arm pilot study. JMIR Ment Health.

[60] Pan JY, Carlbring P, Lu L Efficacy of internet-based cognitive behavioral therapy for Hong Kong university students: a randomized controlled trial. Res Soc Work Pract.

[61] Ha SW, Kim J (2020). Designing a scalable, accessible, and effective mobile app based solution for common mental health problems. Int J Hum-Comput Interact.

[62] Kroenke K, Spitzer RL, Williams JB (2001). The PHQ-9: validity of a brief depression severity measure. J Gen Intern Med.

[63] Park SJ, Choi HR, Choi JH, Kim KW, Hong JP (2010). Reliability and validity of the Korean version of the Patient Health Questionnaire-9 (PHQ-9). Anxiety Mood.

[64] An JY, Seo ER, Lim KH, Shin JH, Kim JB (2013). Standardization of the Korean version of screening tool for depression (Patient Health Questionnaire-9, PHQ-9). J Korean Soc Biol Ther Psychiatry.

[65] Beck AT, Steer RA, Depression B (1996). Inventory—2nd Edition Manual.

[66] Kim JH, Lee EH, Hwang ST, Hong SH (2015). Korean Version of the Beck Depression Inventory-II Manual.

[67] Sung HM, Kim JB, Park YN, Bae DS, Lee SH, Ahn HY (2008). A study on the reliability and the validity of Korean version of the Beck Depression Inventory-II (BDI-II). J Korean Soc Biol Psychiatry.

[68] Hollon SD, Kendall PC (1980). Cognitive self-statements in depression: development of an automatic thoughts questionnaire. Cogn Ther Res.

[69] Kwon SM, Yoon HK (1994). Development and application of the Korean version of the Automatic Thoughts Questionnaire. Stud Res.

[70] Heo S, Kim J (2020). Development and validation of short form of the Korean version of the Automatic Thoughts Questionnaire-Negative. HSS21.

[71] Heo S, Kim J (2020). A validation of the Korean Version of the Positive Subscale of the Automatic Thoughts Questionnaire-Revised (K-ATQ-RP). HSS21.

[72] Kendall PC, Howard BL, Hays RC (1989). Self-referent speech and psychopathology: the balance of positive and negative thinking. Cogn Ther Res.

[73] Baker RW, Siryk B (1984). Measuring adjustment to college. J Couns Psychol.

[74] Lee YJ (1999). The Non-Residential Students’ Adaptation to College Life and Career Plans.

[75] Stoyanov SR, Hides L, Kavanagh DJ, Zelenko O, Tjondronegoro D, Mani M (2015). Mobile App Rating Scale: a new tool for assessing the quality of health mobile apps. JMIR mHealth uHealth.

[76] Gregoire J (2018). ITC guidelines for translating and adapting tests (second edition). Int J Test.

[77] Chung KM, Suh YJ, Chin S (2022). A pilot study testing the efficacy of dCBT in patients with cancer experiencing sleep problems. Front Psychol.

[78] Mind booster green. iApp Store.

[79] Jacobson NS, Truax P (1991). Clinical significance: a statistical approach to defining meaningful change in psychotherapy research. J Consult Clin Psychol.

[80] Hubbard AE, Ahern J, Fleischer NL (2010). To GEE or not to GEE: comparing population average and mixed models for estimating the associations between neighborhood risk factors and health. Epidemiology.

[81] Beck JS (2020). Cognitive Behavior Therapy: Basics and Beyond.

[82] Cristea IA, Kok RN, Cuijpers P (2015). Efficacy of cognitive bias modification interventions in anxiety and depression: meta-analysis. Br J Psychiatry.

[83] Forand NR, Barnett JG, Strunk DR, Hindiyeh MU, Feinberg JE, Keefe JR (2018). Efficacy of guided iCBT for depression and mediation of change by cognitive skill acquisition. Behav Ther.

[84] Mogoaşe C, David D, Koster EHW (2014). Clinical efficacy of attentional bias modification procedures: an updated meta-analysis. J Clin Psychol.

[85] Wu A, Scult MA, Barnes ED, Betancourt JA, Falk A, Gunning FM (2021). Smartphone apps for depression and anxiety: a systematic review and meta-analysis of techniques to increase engagement. NPJ Digital Med.

[86] Domhardt M, Steubl L, Boettcher J (2021). Mediators and mechanisms of change in internet- and mobile-based interventions for depression: a systematic review. Clin Psychol Rev.

[87] Mogoașe C, Cobeanu O, David O, Giosan C, Szentagotai A (2017). Internet-based psychotherapy for adult depression: what about the mechanisms of change?. J Clin Psychol.

[88] Christensen H, Griffiths KM, Farrer L (2009). Adherence in internet interventions for anxiety and depression. J Med Internet Res.

[89] Kelders SM, Kok RN, Ossebaard HC, Van Gemert-Pijnen JEWC (2012). Persuasive system design does matter: a systematic review of adherence to web-based interventions. J Med Internet Res.

[90] Hamid A, Arshad R, Shahid S (2022). What are you thinking? using CBT and storytelling to improve mental health among college students.

[91] Busselle R, Bilandzic H (2009). Measuring narrative engagement. Media Psychol.

[92] Brown M, O’Neill N, van Woerden H, Eslambolchilar P, Jones M, John A (2016). Gamification and adherence to web-based mental health interventions: a systematic review. JMIR Ment Health.

[93] Xu L, Shi H, Shen M (2022). The effects of mHealth-based gamification interventions on participation in physical activity: systematic review. JMIR Mhealth Uhealth.

[94] Lau N, O’Daffer A, Yi-Frazier JP, Rosenberg AR (2021). Popular evidence-based commercial mental health apps: analysis of engagement, functionality, aesthetics, and information quality. JMIR Mhealth Uhealth.

[95] Six SG, Byrne KA, Tibbett TP, Pericot-Valverde I (2021). Examining the effectiveness of gamification in mental health apps for depression: systematic review and meta-analysis. JMIR Ment Health.

